# The Effect of Vitamin D Treatment on Serum Adiponectin Levels in Children with Vitamin D Deficiency Rickets

**DOI:** 10.4274/jcrpe.v1i6.262

**Published:** 2010-12-08

**Authors:** Behzat Özkan, Hakan Döneray, Halil Keskin

**Affiliations:** 1 Atatürk University, Faculty of Medicine, Department of Pediatrics, Division of Pediatric Endocrinology and Metabolism, Erzurum, Turkey; +90 532 513 22 99ozkan.behzat@gmail.comAtatürk Üniversitesi, Yakutiye Araştırma Hastanesi Çocuk Servisi, 25100 Erzurum

**Keywords:** treatment, Vitamin D, Adiponectin, rickets

## Abstract

**Objective**: Adiponectin and its receptors are known to be expressed in osteoblasts and may have important functions in normal bone cells. The aim of this study was to investigate the effect of vitamin D therapy on serum adiponectin levels in children with vitamin D deficiency rickets (VDDR).

**Methods**: 21 patients with VDDR were included in the study. Patients were treated with 300,000 U D3 (IM) and calcium lactate (50mg/kg/ day, PO, for 10 days). Anthropometric parameters and serum biochemical markers including calcium (Ca), phosphorus (P), alkaline phosphatase (ALP), intact parathormone (iPTH), 25-hydroxyvitamin D (25(OH)D), and adiponectin levels were measured before and after one month of therapy.

**Results**: Weight and length, but not BMI, increased significantly after treatment. Serum 25(OH)D level increased significantly after treatment, while serum adiponectin level decreased (4.21±1.84 vs 52.73±17.63 ng/ml, p<0.000; 150.1±66.14 vs 84.29±9.06 mg/ml, p<0.000, respectively). No significant correlations were found between serum adiponectin and 25(OH)D levels before and after treatment or between delta adiponectin concentrations and delta 25(OH)D levels.

**Conclusion**: Serum adiponectin levels are increased in patients with VDDR, a finding which is probably related to increased osteoblastic activity.

**Conflict of interest:**None declared.

## INTRODUCTION

Adiponectin, a 244-amino acid protein and abundantly present in the plasma, is synthesized and secreted exclusively by the adipose tissue (1, 2). Adipocytes and osteoblasts are both of mesodermal origin (2). Osteoblasts have been de monstrated to express adiponectin in lower levels than in subcutaneous adipose tissue (2, 3, 4). In addition, adiponectin receptors are present in human osteoblasts, suggesting that it has paracrine and endocrine effects on bone-forming cells (5).

Rickets is characterized by a failure in mineralisation of osteoid tissue (6). Vitamin D deficiency (VDD) is the predominant cause of rickets and is associated with an increase in osteoblastic activity and possibly with serum adiponectin levels (7, 8). The aim of this study was to investigate the effect of vitamin D treatment on serum adiponectin levels in children with vitamin D deficiency rickets (VDDR).

## METHODS

The study was approved by the Ethical Committee of Ataturk University Faculty of Medicine and written informed consent was obtained from the parents of all participants. This longitudinal study was conducted between March 2007 and February 2008. The study group consisted of otherwise healthy children aged 2-24 months whose body weight and height values were between 3^rd^ and 97^th^ percentiles, and who were diagnosed as VDDR. Clinical rickets was diagnosed in children who showed two or more of the following findings: craniotabes (in infants over 2 months), bilateral widened wrists, frontal bossing, bowing of the legs, pathologic fractures, hypocalcemic tetany, hypocalcemic convulsions, and Harrison’s sulcus. Infants with familial forms of rickets and those with secondary VDD due to kidney, liver and gastrointestinal system diseases were excluded from the study. 

The anthropometric indices of the patients were recorded before and after therapy. Weight was measured using an electronic scale (Seca Model 770, Hamburg, Germany). Length (±0.1 cm) was measured using a body-length measurer by a paediatrician. Body mass index (BMI) was calculated as body weight (kg)/square of length (m2). Radiological and laboratory investigations were carried out in all cases diagnosed as VDDR. Radiological evidence of rickets by radiography of the left wrist included two or more of the following signs: generalized osteopenia, fraying and cupping of the distal ends of the radius or ulna. To confirm the diagnosis of VDDR biochemically, serum calcium (Ca), phosphorus (P), alkaline phosphatase (ALP), serum intact parathyroid hormone (iPTH), and 25-hydroxyvitamin D (25(OH)D) were measured in all patients. VDD was defined as a serum 25(OH)D level less than 10 ng/ml (9).

All patients were treated with an intramuscular single dose (300.000 IU) of vitamin D3, and 50 mg/kg/day of elementary calcium lactate administered orally for ten days. The patients were invited to the clinic one month after the initiation of therapy. Serum Ca, P, ALP, iPTH, and 25(OH)D were re-measured and radiography of the left wrists were re-evaluated. If the serum Ca, P, and 25(OH)D concentrations increased while serum iPTH and ALP concentrations decreased compared to pre-treatment values, and if a calcification zone along the metaphyseal-diaphyseal border was detected radiologically, the patient was considered to be healed.

**Measurement of serum adiponectin levels, and biochemical and hormonal parameters**

Blood samples were obtained before and after treatment. Biochemical tests were studied immediately. For hormone measurements, serum samples were transferred into Eppendorf tubes and stored at -20 oC until assayed. Adiponectin was measured by ELISA using the kits and protocol from ORGENIUM Laboratories, Inc. (AviBion Human Adiponectin (Acrp30) ELISA Kit, Helsinki, Finland). Serum Ca, P, and ALP levels were measured using the calorimetric method. Serum iPTH was determined with chemiluminescence enzyme immunoassay method using the iPTH kit (IMMULITE 2000 Intact PTH Kit, DPC Co., USA). Serum 25(OH)D level was determined by competitive binding RIA (Gamma-B, 25(OH)D RIA, Immunodiagnostic Systems, UK). 

All the calculations were made by using SPSS (version 15.0, Chicago, IL, USA) for Windows. The Wilcoxon-Smirnov test was used for the significance level of the differences between longitudinal patterns. Correlations between two variables were tested by Pearson’s correlation coefficient. The results were expressed as mean±SD; p<0.05 was considered statistically significant.

## RESULTS

Twenty-one cases with rickets completed the study. Mean follow-up duration was 28±2 (25-32) days. The median chronological age at referral was 7.3 (2-16) months. Anthropometric parameters before and after therapy are shown in Table 1. Weight and height increased significantly after treatment, but there was no change in BMI. Biochemical markers measured before and after treatment are shown in Table 2. No significant correlations were found between serum adiponectin and 25(OH)D levels before and after treatment, or between delta adiponectin concentrations and delta 25(OH)D levels. Serum adiponectin concentration was positively correlated with mean ALP level in patients with VDDR before treatment (r=0.722, p<0.001).

**Table 1:**
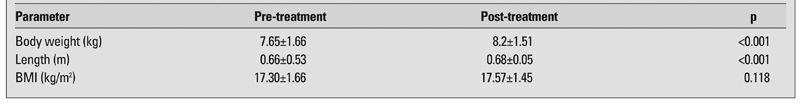
Anthropometric indices of the patients before and after treatment (mean±SD)

**Table 2:**
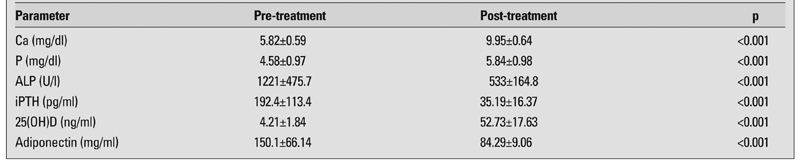
Changes in the biochemical and hormonal parameters of the patients during the study period (mean±SD)

## DISCUSSION

To our knowledge, this is the first study investigating the relationship between serum 25(OH)D and adiponectin concentration in children diagnosed with VDDR. We found that serum adiponectin levels decreased significantly in cases with VDDR on treatment. 

VDDR, a growing bone disease, is mostly encountered in the developing regions of the world (8, 10). The etiology of nutritional rickets (NR) has a spectrum range from isolated VDD to isolated Ca deficiency (11, 12). In Turkey, VDD is the cause of almost all NR cases (13), while in Egypt and Nigeria, the condition is due to calcium insufficiency and/or VDD (14). In this present study, dietary Ca levels of the patients were not measured and all patients with NR were accepted as VDDR.

Both adipocytes and osteoblasts are cells of mesodermal origin (2). Cells of mesenchymal origin share many characteristics in gene expression during differentiation. Even though their phenotypes may differ markedly, mature adipocytes and osteoblasts express and secrete several common factors. This is a sign of close relationship between these cells (15). The experimental results of adiponectin expression and secretion from bone-forming cells demonstrate that adiponectin is not adipocyte specific, and that it may have important functions in normal bone cells (2). This is underlined by the recently reported association between serum adioponectin concentration and bone mineral density (16). It was also reported that adiponectin expression in osteoblasts is much lower than in subcutaneous adipose tissue, an expected finding considering that it is synthesized and secreted exclusively by the adipose tissue (2, 3, 4). In this study, serum adiponectin concentration significantly decreased after treatment, while there was no difference in BMI scores of the cases before and after treatment. This finding suggests that the increased adiponectin level in VDDR may result from bone tissue with increased osteoblastic activity. In this study, it was shown that mean serum adiponectin concentration was positively correlated with mean ALP level that was the sign of the high osteoblastic activity in cases with VDDR before vitamin D therapy. The effect of the vitamin D treatment on the osteoblastic activity, in turn, on the adiponectin level after the treatment of rickets confirm that increased serum 25(OH)D level is associated with decreased osteoblastic activity. In previous studies (1, 5), it was reported that presence of adiponectin receptors in human osteoblasts suggests that adiponectin has paracrine and endocrine effects on bone-forming cells (5). It was also shown that adiponectin increased mRNA expression of ALP and mineralisation activity of mouse osteoblasts, indicating that adiponectin may have a potential to activate osteoblasts (1). 

On the other hand, it was reported in some studies that adiponectin increases the osteoclastic activity, and primary osteoclasts express both adiponectin R1 and adiponectin R2 receptors, suggesting that adiponectin may directly target osteoclast cells (1, 2, 17). Clinical studies show that patients with VDDR have high bone resorption markers indicating high bone turn over (7, 18). In the present study, serum adiponectin concentration before treatment was significantly high and decreased on therapy. 

In conclusion, this is the first study investigating the relation between serum 25(OH)D and adiponectin concentration in children diagnosed with VDDR. The results of the study show that serum adiponectin levels are increased in patients with VDDR, a finding which is probably related to increased osteoblastic activity. The regulation of bone metabolism by adipokines is still largely unknown and future studies are needed to better understand the relationship between adipokines and bone.
